# Mild Sensory Stimulation Protects the Aged Rodent From Cortical Ischemic Stroke After Permanent Middle Cerebral Artery Occlusion

**DOI:** 10.1161/JAHA.112.001255

**Published:** 2012-08-24

**Authors:** Christopher C. Lay, Melissa F. Davis, Cynthia H. Chen-Bee, Ron D. Frostig

**Affiliations:** 1 Department of Neurobiology and Behavior, Irvine, CA (C.C.L., M.F.D., C.H.C.-B., R.D.F.); 2 Department of Biomedical Engineering, Irvine, CA (R.D.F.); 3 The Center for the Neurobiology of Learning and Memory, Irvine, CA (C.C.L., M.F.D., C.H.C.-B., R.D.F.); 4 The Center for Hearing Research, University of California, Irvine, CA (C.C.L, R.D.F.)

**Keywords:** aging, imaging, ischemia, stroke

## Abstract

**Background:**

Accumulated research has shown that the older adult brain is significantly more vulnerable to stroke than the young adult brain. Although recent evidence in young adult rats demonstrates that single-whisker stimulation can result in complete protection from ischemic damage after permanent middle cerebral artery occlusion (pMCAO), it remains unclear whether the same treatment would be effective in older animals.

**Methods and Results:**

Aged rats (21 to 24 months of age) underwent pMCAO and subsequently were divided into “treated” and “untreated” groups. Treated aged rats received intermittent single-whisker stimulation during a 120-minute period immediately after pMCAO, whereas untreated aged rats did not. These animals were assessed using a battery of behavioral tests 1 week before and 1 week after pMCAO, after which their brains were stained for infarct. An additional treated aged group and a treated young adult group also were imaged with functional imaging. Results demonstrated that the recovery of treated aged animals was indistinguishable from that of the treated young adult animals. Treated aged rats had fully intact sensorimotor behavior and no infarct, whereas untreated aged rats were impaired and sustained cortical infarct.

**Conclusions:**

Taken together, our results confirm that single-whisker stimulation is protective in an aged rodent pMCAO model, despite age-associated stroke vulnerability. These findings further suggest potential for translation to the more clinically relevant older adult human population. **(*J Am Heart Assoc*. 2012;1:e001255 doi: 10.1161/JAHA.112.001255.)**

## Introduction

Stroke is the most prevalent cause of age-related brain injuries resulting in permanent physical and mental disability.^1–2^ Old age is associated with an enhanced susceptibility to stroke, poor recovery from infarct,^1,3^ and the decreased effectiveness of neuroprotective therapy in both humans and rats.^1,4–6^

Our laboratory has previously demonstrated that sensory stimulation (intermittent mechanical single-whisker stimulation) delivered within 1 hour (and in most cases within 2 hours) after permanent middle cerebral artery occlusion (pMCAO) is completely protective in a young adult model (3- to 4-month-old rats). The stimulus used was exceedingly mild: Treatment consisted of 4.27 minutes of 1-second, 5-Hz, 9° deflections of a single whisker intermittently during a 120-minute treatment period. This treatment resulted in the gradual recovery of cortical function and reperfusion via collateral vessels during the treatment period itself. These results were confirmed by multiple techniques, including functional imaging, blood flow imaging, electrophysiological recording, behavioral assessment, and histology.^7–8^ Functional imaging results revealed that, after pMCAO, stimulation initiated a gradual return of functional response, which recovered to baseline level during the 120-minute treatment period. Neuronal recording, functional imaging, and blood flow imaging data demonstrated that the cortex was functioning at or above baseline levels at 24 hours after pMCAO. Additionally, behavioral and histological assessment at 7 days after pMCAO confirmed that rats had no sensorimotor deficits and no infarct.^8^ (Untreated animals and animals treated >2 hours after pMCAO, however, lost cortical function and suffered sensorimotor deficits and infarct).

**Figure 1.**
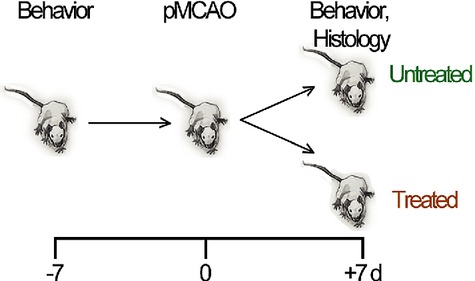
Experimental schematic. Rats 21 to 24 months of age underwent baseline behavioral assessment, followed 1 week later by pMCAO; behavior testing and histological assay were conducted 1 week after pMCAO.

Could the same mild whisker-stimulation treatment protect the aged rat, despite the increased vulnerability to ischemic damage? To answer this question, older rats (21 to 24 months old; equivalent to 60 to 65 years of age in humans)^9^ underwent the same pMCAO and whisker-stimulation treatment as their younger counterparts from the previous studies.^7–8^ Aged animals were assessed for baseline sensorimotor capacity by using 3 tests: the Bederson neurological scale, forepaw-guided exploration, and whisker-guided exploration. One week later, all rats underwent pMCAO and then were placed randomly into 1 of 2 groups: “treated” rats that received stimulation treatment immediately after pMCAO and “untreated” control rats that never received stimulation treatment. One week after pMCAO, both groups underwent behavioral reassessment and postmortem histological analysis (Figure 1). Treated aged rats were unimpaired on all 3 sensorimotor tasks and did not sustain infarct. Untreated aged controls, however, demonstrated sensorimotor impairment and sustained infarct.

An additional group of treated aged rats and a group of treated young adult rats underwent functional imaging before treatment, during treatment, and 24 hours after pMCAO to assess degree and rate of functional recovery in protected aged animals versus protected young adults. Surprisingly, aged rats recovered cortical function at the same rate as young adults.

In summary, we confirmed that single-whisker stimulation completely protected sensorimotor behavior and cortical function in aged rats as it had in young adults, and according to 2,3,5-triphenyltetrazolium chloride (TTC) staining at 7 days after pMCAO, also completely protected cortical structure. (Although we acknowledge the possibility that macrophage invasion at 7 days could have caused masking of some small amount of infarct, the animals were at worst protected from the infarct observed in untreated animals and at best completely protected from infarct.^10^) Surprisingly, the cortex of older rats seemed equally amenable to protection via stimulation treatment when compared to the cortex of young adult rats. These results suggest that the capacity of the cortex to be completely protected from stroke by mild sensory stimulation is not diminished by aging brain tissue or associated vascular degeneration.

## Methods

### Subjects and Surgical Preparation

All procedures were in compliance with National Institutes of Health guidelines and were approved by the University of California Irvine Animal Care and Use Committee (protocol No. 1997-1608; assurance ID No. A3416.01).

Seven male Sprague Dawley rats (3 to 4 months of age) and 35 male Fischer 344 rats (21 to 24 months of age) were kept under standard laboratory conditions. (Different strains of rats were used because the National Institute on Aging breeds only aged Fischer rats). Body weights ranged from 350 to 550 g for the older animals and 300 to 400 g for the young animals. Rats had free access to food and water. At the beginning of each experiment, rats were injected with a pentobarbital bolus (55 mg/kg body weight), followed by an injection of atropine (0.05 mg/kg body weight). Supplemental injections of pentobarbital (27.5 mg/kg body weight) were given as necessary.

Similar to previous research that reported an elevated rate of post–middle cerebral artery (MCA) occlusion death in Fischer 344 rats,^11^ 8 aged Fischer rats had to be excluded from this study because of hemorrhage or death during or soon after surgical procedure. Additionally, 2 were disqualified because of too little exploratory activity during behavioral testing. Of the remaining 25 aged rats, 10 rats were used in the treated behavioral group, 8 rats were used in the untreated behavioral group, and 7 rats were used in the functional imaging group.

### Permanent Middle Cerebral Artery Occlusion

Ischemic conditions were achieved via surgical occlusion and transection of the M1 segment (just distal to lenticulostriate branching) of the left MCA such that only MCA cortical branches were affected and thus only cortical infarct (no subcortical damage) was expected.^12–13^ The skull and dura were removed carefully from a 2×2-mm “surgical window” just anterior and lateral to the imaging window (over the occlusion location; the M1 segment just distal to the MCA's lenticulostriate branching), and a half-curve reverse-cutting suture needle and thread (4-0 silk) were passed through the pial layer of the meninges, below the MCA and above the cortical surface. A double ligature was tied and tightened around the MCA, and the vessel then was transected (completely severed) between the 2 knots. Experiments were terminated if there was any sign of bleeding from the MCA or if there were obvious arterial abnormalities or malformations.^14^

### Stimulation Treatment

Whisker stimulation consisted of 1 second of 5-Hz mechanical deflections of a single whisker (whisker C2, contralateral to the ischemic hemisphere) by 9° in the rostral–caudal direction. This stimulation was delivered intermittently (with random intervals averaging 21 seconds) 256 times, totaling 4.27 minutes of stimulation, over the course of 2 hours. The 256 stimulus exposures were delivered in four 64-trial blocks to allow for (optional) functional imaging and anesthetic administration. The remainder of the 2-hour treatment period was taken up by anesthetic administration and assessment of the animal's condition in between stimulus blocks. All subjects remained anesthetized throughout the treatment period.

### Sensorimotor Behavior

To evaluate changes in neurological function associated with ischemia, sensorimotor behavior was assessed 7 days before and 7 days after pMCAO on 3 tasks sensitive to stroke behavioral deficit: the Bederson neurological scale,^15^ forepaw-guided exploration,^16^ and whisker-guided exploration.^17–18^ An observer blind to the rats’ experimental conditions performed testing and analysis of behavioral data.

For Bederson neurological scale assessment, rats first were held by the base of the tail and suspended 2.5 meters above the floor for 10 seconds to determine whether the rats withdrew the affected limb, a recognized sign of ischemic injury. Next, rats were placed into large cylindrical chambers, were allowed to roam freely for 5 minutes, and were observed for signs of spontaneous circling behavior, difficulty with gait, and difficulty remaining upright. Animals were scored on a 0-to-4 scale, as follows: 0, normal movement; 1, failure to extend forepaw contralateral to infarct; 2, circling locomotive behavior; 3, falling to the side of infarct; and 4, lack of spontaneous movement or stupor associated with very severe cortical and subcortical lesions.^6^

Forepaw-guided exploration was assessed by placing each rat in a testing cylinder (20 cm in diameter and 45 cm in height). Wall touch after rearing, using the left forepaw, right forepaw, or both paws together, was recorded for 5 minutes.

Whisker-guided exploration was evaluated by placing each rat in a 25-cm-wide rectangular track (120×80 cm, outer diameter). Rats were allowed 10 seconds to acclimate before the 5-minute testing session. Whisker-guided exploration was defined as number of seconds spent touching the walls of the rectangular track with each set of whiskers while locomoting.^17^

A forepaw or whisker use asymmetry score was calculated (*r* − *l*)/(*r* + *l*), where “*r*” and “*l*” represent right and left whisker set or right and left forepaw, respectively). A negative score signifies a subject's preference to explore with the left forepaw or whisker. Healthy rats use paws and whisker pads symmetrically, whereas rats with unilateral cortical damage display asymmetric use.^16,18^

Because the Bederson neurological score is a categorical assessment, the nonparametric Mann-Whitney *U* test was used to compare between groups at baseline and to compare the difference in score before and after pMCAO between groups. Also, within each group, the Wilcoxon signed rank test was used to compare before and after pMCAO.

Forepaw-guided asymmetry, whisker-guided asymmetry, overall forepaw exploration, and overall whisker exploration were assessed with repeated-measures analysis of variance (ANOVA). Two ANOVAs were conducted to determine if (1) forepaw asymmetry or (2) whisker asymmetry was affected by treatment after pMCAO. The second pair of ANOVAs was conducted to determine whether there were differences in locomotor activity during either forepaw-guided exploration or whisker-guided exploration that could confound the exploration asymmetry test results. In total, 4 separate repeated-measures ANOVAs were performed and reported, describing behavioral asymmetry and overall exploration activity before and after pMCAO. Respective contrasts then were performed to identify which postocclusion values were significantly different from baseline. Alpha level was set to 0.05, and Bonferroni adjustments were applied to account for multiple contrasts.

### Histology (TTC Staining for Infarct)

Rats were euthanized, and their brains were removed, sectioned into 2-mm coronal slices, and incubated in 2% TTC at 37°C for 20 minutes in the dark.^15^ TTC is enzymatically reduced in active mitochondria, producing formazan (a bright red byproduct). Unstained (white) areas are thus indicative of infarct.^19–20^ The TTC-stained sections were photographed with a digital camera, and the total infarct volume was determined by multiplying the infarct area of each slice by the thickness and summing across slices. An observer blind to the rats’ experimental conditions performed this volume calculation.

### Intrinsic Signal Optical Imaging and Analysis

We used the functional imaging technique intrinsic signal optical imaging (ISOI) to assess whisker functional representation (WFR) before pMCAO, every 30 minutes during the treatment period, and 24 hours after pMCAO. ISOI has been used extensively to provide high-spatial-resolution maps of stimulus-evoked hemodynamic-related signals as an indirect means to image the functional organization of the cortex and examine how these signals contribute to brain function.^21–23^ Although these associations are still debated, the initial dip phase of the WFR is generally associated with evoked neuronal activity and the overshoot phase with blood flow response (for a recent review of ISOI, see Frostig and Chen-Bee, 2009^24^).

A detailed description of ISOI^21–23^ data acquisition and analysis also can be found elsewhere.^25–26^ Briefly, the cortex was illuminated with a red light-emitting diode (635 nm maximum wavelength with full width at half height of 15 nm), and a charge-coupled device camera was used for imaging. During each 15-second trial, 1.5 second of prestimulus data followed by 13.5 seconds of poststimulus onset data were collected, with a 6±5-second random intertrial interval. Stimulus consisted of a single whisker (whisker C2) being deflected by ≍9° in the rostral–caudal direction at a rate of 5 Hz for a total stimulus duration of 1 second. Data were collected in blocks of 64 stimulation trials over periods of about 30 minutes each. Ratio images were created from calculating fractional change values, as described previously.^27–28^ The first 2 phases of WFR were analyzed: the initial dip and the overshoot. The ratio image containing the maximal areal extent for each of the 2 phases was Gaussian filtered, and the areal extent was quantified at a threshold level of 2.5×10^−4^ fractional change away from zero. Peak amplitude was quantified in fractional change units of the peak activity pixel for each of the 2 intrinsic signal phases.

For statistical analysis, ANOVAs were used with an α-level of significance set at *P*<0.05. An initial set of 4 repeated-measures ANOVAs was used to compare between young and aged animal groups at baseline and during 24-hour reassessment imaging. This initial series included analysis of the initial dip (area and amplitude) and overshoot (area and amplitude) phases of the WFR.

A second set of 4 repeated-measures ANOVAs was used to compare between young and aged animal groups during the post-pMCAO treatment period. Post-pMCAO data were converted to difference score values (postocclusion – baseline), and a constant was added to the difference values, which then were transformed with a square root function to better satisfy the assumptions of an ANOVA (normal distribution for each subgroup of values, homogeneity of variance across means, independence between variance and means). Alpha level was set to 0.05, and Bonferroni adjustments were applied to account for multiple contrasts.

## Results

### Single-Whisker Stimulation Treatment Protects Sensorimotor Behavior and Cortical Structure According to TTC at 7 Days After pMCAO in an Aged Model of Ischemic Stroke

Two groups of aged rats received pMCAO and underwent behavioral assessment twice (before and 7 days after undergoing pMCAO). One group (n=10) received stimulation treatment immediately after pMCAO, whereas the other group (n=8) did not. Behavior was assessed with 3 tests: Bederson neurological scale, forepaw-guided exploration, and whisker-guided exploration. Mann-Whitney *U* tests were performed on the Bederson data to compare between groups (treated versus untreated); one test was performed on data obtained before pMCAO, and another test was performed on the within-subjects difference in Bederson scale obtained before pMCAO versus 7 days after pMCAO. A repeated-measures ANOVA with 1 within-subjects variable (time of assessment; before versus after) and 1 between-subjects variable (group of rats; treated versus untreated) was performed on the forepaw exploration data; the ANOVA was followed by specific contrasts for testing additional hypotheses. A repeated-measures ANOVA with specific contrasts was performed similarly on the whisker-guided exploration data.

**Figure 2.**
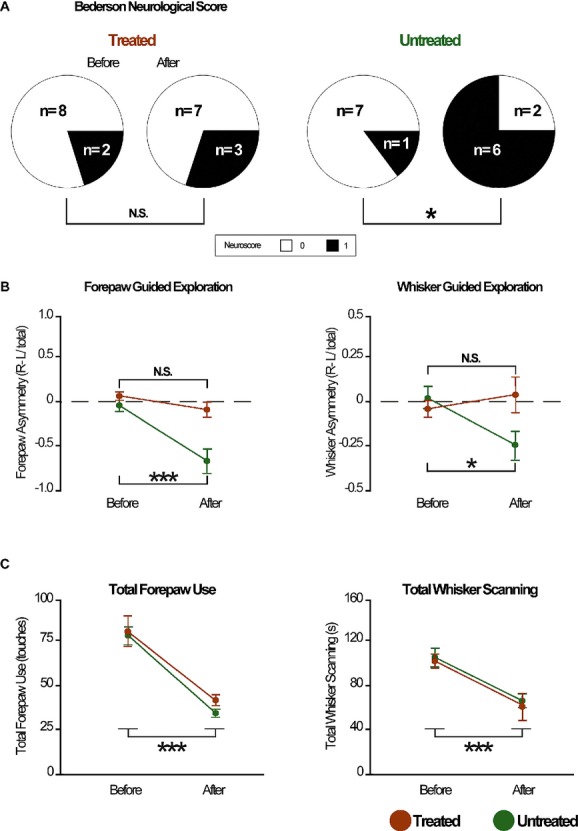
Stimulation treatment protects sensorimotor behavior in aged rats. Testing was conducted 1 week before and 1 week after pMCAO. A, Neurological scores according to the Bederson scale. Pie charts represent the number of rats with the corresponding neurological score (see key). Although the Bederson scale ranges from 0 to 4, animals in this study scored between 0 and 1 only. B, Forepaw-guided (left) and whisker-guided (right) asymmetry scores before and after pMCAO. Horizontal line indicates “0,” or no asymmetry. C, Total amount of forepaw-guided exploration before and after pMCAO (left) and total amount of whisker-guided exploration scanning before and after pMCAO (right). Asterisk indicates a significant difference from baseline; *P<0.05 and ***P<0.001.

Before pMCAO, the distribution of Bederson scale values was similar between the 2 rat groups, with the majority of values being a scale of zero, indicating no deficit (see Figure 2A); no significant difference was found between the 2 rat groups (Mann-Whitney *U*=49, n_1_=10, n_2_=8, *P*=0.61). In contrast, the rat groups differed with regard to the change in Bederson scale before pMCAO and 7 days after pMCAO. For untreated rats, the distribution of Bederson scale values 7 days after pMCAO contained more values of 1 (failure to extend forepaw contralateral to pMCAO) than before pMCAO, whereas for treated rats, the distribution of Bederson scale values remained the same (Figure 2A). Although a Mann-Whitney *U* test performed on the change in Bederson scale did not find this difference between rat groups significant (Mann-Whitney *U*=21.5, n_1_=10, n_2_=8, *P*=0.06), Wilcoxon signed rank tests performed on each rat group found that the Bederson scale values were different before and after pMCAO for the untreated rat group (Wilcoxon *Z*=2.24, *P*=0.03) but not for the treated rat group (Wilcoxon *Z*=0.58, *P*=0.56).

Complementary results were obtained for the forepaw-guided exploration (Figure 2B, left) and the whisker-guided exploration (Figure 2B, right). Before pMCAO, untreated and treated rats similarly exhibited no preference in forepaw or whisker use between the left and right sides. In contrast, 7 days after pMCAO, untreated rats exhibited exploratory behavior with increased preference to use the forepaw and whiskers on the left side (ipsilateral to pMCAO), whereas treated rats exhibited no side preference in forepaw or whisker use. In support of these observations, a repeated-measures ANOVA performed on the forepaw-guided exploration data found an interaction between treatment group and time of assessment (F_1,16_=11.19, *P*=0.004). Additional contrasts found no significant difference between treatment groups before pMCAO (F_1,16_=2.93, *P*=0.106) and instead found a significant difference before and after pMCAO for untreated rats (F_1,16_=34.77, *P*=2×10^−5^) but not for rats that received treatment (F_1,16_=2.48, *P*=0.135). For the whisker-guided exploration data, a repeated-measures ANOVA followed by additional contrasts also found a significant interaction between treatment group and time of assessment (F_1,16_=5.21, *P*=0.036), no significant difference between treated and untreated rats before pMCAO (F_1,16_=0.67, *P*=0.423), and a significant difference before and after pMCAO for untreated rats (F_1,16_=5.95, *P*=0.027) but not for rats that received treatment (F_1,16_=0.49, *P*=0.495).

Total time spent exploring with forepaws (Figure 2C, left) or whiskers (Figure 2C, right) before pMCAO and 7 days after pMCAO also was compared between the 2 treatment groups to determine whether any differences occurred in overall locomotion. For both types of exploration, the 2 treatment groups exhibited similar levels of total exploration before pMCAO and 7 days after pMCAO, although for both groups, the total amount of exploration was observed to decrease 7 days after pMCAO as compared to before pMCAO, likely because of habituation.^16,29^ In line with these observations, repeated-measures ANOVAs performed on the forepaw and whisker total exploration data, respectively, found the main effect Time of Assessment to be significant (F_1,16_=56.79, *P*=1×10^−6^; F_1,16_=33.08, *P*=3×10^−5^) but not the main effect Treatment Group (F_1,16_=1.50, *P*=0.239; F_1,16_=0.12, *P*=0.736) or their interaction (F_1,16_=0.17, *P*=0.686; F_1,16_=0.00, *P*=0.974).

**Figure 3.**
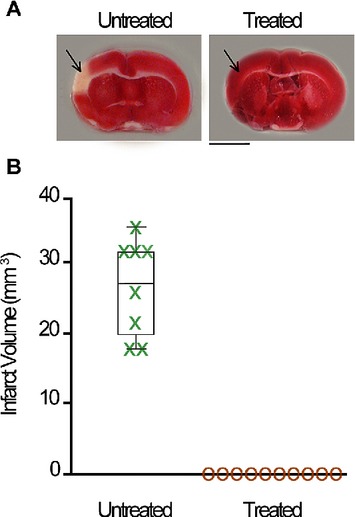
Stimulation treatment protects cortical structure in aged rats. A, Representative coronal sections from an untreated (left) and a treated rat (right) with TTC assay for infarct. Red staining indicates healthy tissue; lack of staining indicates ischemic infarct. Arrows point toward MCA blood supply territory. Scale bar = 5 mm. B, Box-and-whisker plot of infarct volume (mm^3^), with individual subjects plotted for untreated (green) aged subjects and treated (gold) aged groups.

Seven days after pMCAO (after final behavioral assessment), the brains from the 2 aged rat groups underwent histological assessment for infarct presence (Figure 3). All untreated rats (n=8) were found to have sustained cortical infarcts (mean±SEM, 27.6±2.4 mm^3^), whereas none of the treated rats (n=10) had sustained any infarct.

To summarize, treated aged rats displayed sensorimotor behaviors 7 days after pMCAO that were indistinguishable from baseline performance, and they sustained no infarct. In contrast, untreated aged rats were impaired on all 3 sensorimotor behavior tests, and they sustained infarct. These results extend our previous findings by demonstrating that the ability of whisker stimulation to completely protect the cortex from ischemic injury can be extended to the aged cortex.

### Single-Whisker Stimulation Initiates a Return of Cortical Function That Is Completed During the Treatment Period, Irrespective of Animals’ Age

**Figure 4.**
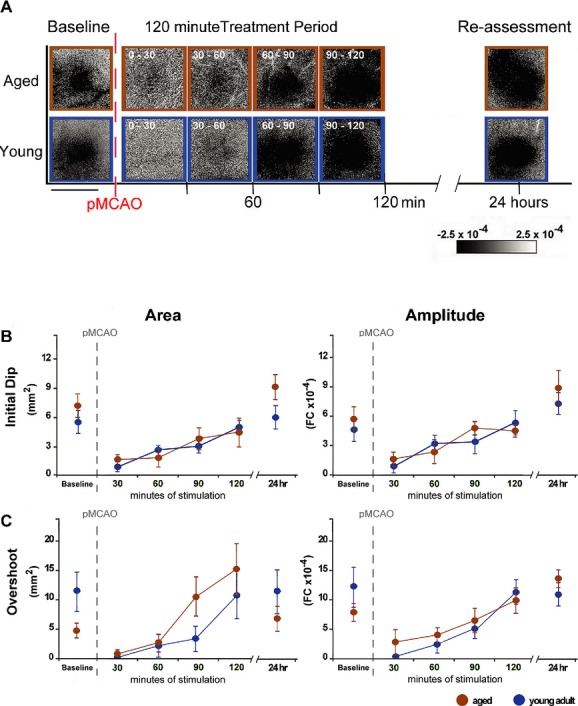
Return of cortical function in aged rats is equivalent to that in young adult rats. A, Representative data from ISOI of the initial dip in aged and young adult representative animals that underwent pMCAO and received whisker-stimulation treatment immediately after occlusion. ISOI was conducted before, during, and 24 hours after pMCAO and whisker-stimulation treatment. Both young adult and aged rats regained evoked functional response comparable to baseline after 90 minutes of whisker-stimulation treatment. Linear grayscale bar indicates intrinsic signal amplitude (fractional change [FC] ×10^−4^). Black and white streaks correspond to large surface blood vessels. Scale bar = 5 mm. B and C, Group baseline is plotted with 120 minutes of postocclusion stimulus period data and 24-hour reassessment data for each graph. Means and standard errors are provided for the area (left) and amplitude (right) of the ipsi-ischemic C2 initial dip (B) and overshoot (C) phases of WFR before and after pMCAO.

In another set of experiments, 2 groups of rats received pMCAO, followed immediately by whisker-stimulation treatment, and underwent assessment of cortical function with ISOI at multiple time points: before pMCAO and 24 hours after pMCAO to determine whether cortical function had been recovered with treatment, as well as 4 time points spaced at equal intervals during the treatment period itself to characterize the time course of recovery (Figure 4A). One group consisted of aged rats (n=7), and the other consisted of young adult rats (n=7). Cortical function was characterized in terms of area, and amplitude of evoked activity was quantified for the first 2 phases (initial dip, see Figure 4B; overshoot, see Figure 4C) of whisker C2's WFR.

Recovery of cortical function was observed to be the same between aged and young adult rats. Irrespective of the type of measurement being quantified (initial dip area or amplitude, Figure 4B; overshoot area or amplitude, Figure 4C), not only did the 2 age groups exhibit similar values before pMCAO, but they also exhibited a similar recovery of cortical function (reestablishment of baseline values) 24 hours after pMCAO. In line with these observations, a repeated-measures ANOVA with 1 within-subjects variable (time of assessment; before pMCAO versus 24 hours after pMCAO) and 1 between-subjects variable (age of rats; aged versus young adult rats) performed on each of the 4 types of data did not find significant either the main effect Age (F_1,12_=3.16, *P*=0.101; F_1,12_=1.03, *P*=0.330; F_1,12_=3.73, *P*=0.08; F_1,12_=0.40, *P*=0.538) or its interaction with time of assessment (F_1,12_=0.98, *P*=0.342; F_1,12_=0.18, *P*=0.682; F_1,12_=0.19, *P*=0.672; F_1,12_=1.31, *P*=0.275), indicating that both groups of rats were the same before pMCAO and 24 hours after pMCAO. The main effect Time of Assessment, however, was found significant for the initial dip amplitude (F_1,12_=13.41, *P*=0.003), indicating that with treatment, the initial dip amplitude at 24 hours after pMCAO was stronger than baseline levels for both age groups.

The time course of recovery during the treatment period itself also was observed to be the same between aged and young adult rats (Figure 4B and 4C). Irrespective of the measurement being quantified, for both age groups, the time course of recovery was observed to consist of a sharp drop in cortical function at the beginning of the treatment period, followed by a gradual increase over the course of the treatment period and ultimately culminating in a reestablishment of baseline values by the end of the treatment period. For the overshoot area (Figure 4C, left), the values for the aged rats were higher than those for the young adult rats throughout the treatment period, but they were still similar to those for the young adult rats in that they exhibited the same time course profile (ie, sharp drop at the start of the treatment period, followed by a gradual increase leading to reestablishment of baseline values by the end of the treatment period). For all 4 measurements, values collected at all 4 time points were converted to difference scores relative to baseline (before pMCAO) and were transformed with a square root function (after addition of a constant to remove negative values) to satisfy ANOVA assumptions. In line with observations, repeated-measures ANOVAs with 1 within-subjects variable (time of assessment within the treatment period; 4 time points spaced equally apart) and 1 between-subjects variable (age of rats; aged versus young adult rats) performed on 3 of the 4 types of data (initial dip area, Figure 4B, left; initial dip amplitude, Figure 4B, right; and overshoot amplitude, Figure 4C, right) found only the main effect Time of Assessment significant (F_3,36_=12.45, *P*=1×10^−5^; F_3,36_=12.93, *P*=1×10^−5^; F_3,36_=12.31, *P*=1×10^−5^), not the main effect Age (F_1,12_=1.15, *P*=0.305; F_1,12_=0.37, *P*=0.553; F_1,12_=2.08, *P*=0.175) or its interaction with the main effect Time of Assessment (F_3,36_=0.85, *P*=0.474; F_3,36_=1.55, *P*=0.219; F_3,36_=1.10, *P*=0.362). Repeated-measures ANOVA performed on the overshoot area (Figure 4C, left) found the main effect Time of Assessment significant (F_3,36_=12.98, *P*=1×10^−5^), as well as the main effect Age (reflective of the higher values observed for the aged rats; F_1,12_=6.68, *P*=0.024), but not their interaction (F_3,36_=0.68, *P*=0.567).

To summarize, by the conclusion of treatment, both groups exhibited baseline or greater levels of all quantified parameters when assessed 24 hours after pMCAO. Also, both groups exhibited a similar time course of recovery during the treatment itself. These data demonstrate that single-whisker-stimulation treatment initiates a similar recovery, with regard not only to outcome but also to time course, for WFR in the young adult and aged rat cortex.

## Conclusions

Although nearly three quarters of all strokes occur in people over the age of 65 years,^30^ the majority of experimental studies of stroke have been performed in young animals and therefore may not fully model the effects of ischemia in older adult subjects.^4^ In both humans and rodents, old age is associated with an increased likelihood of stroke damage resulting from an ischemic incident, poorer predicted outcome, and a reduction in positive response to treatments.^1,3–6^ Additionally, physical inactivity (an issue in older human populations) directly contributes to many of the risk factors associated with stroke.^31^ As a result, many researchers have argued strongly for increased use of aged animals in preclinical stroke experimentation.^32–35^ In line with these recommendations, in the present study, we used aged subjects that were individually housed in standard scientific cages for the entirety of their lives, which limited them to an unnaturally sedentary lifestyle. In the present study, we compared results in our aged Fischer rat subjects to results in young adult Sprague Dawley rats to confirm that mild sensory stimulation delivered immediately after ischemic onset confers complete protection from ischemic damage (as assessed via histology, behavior, and functional imaging at 7 days after pMCAO) to aged rats, which more appropriately model the human population most vulnerable to stroke. The major finding of the present project was that aged subjects could indeed also be protected, as described above.

The most clinically relevant means of inducing ischemia and the best animal model in terms of clinical relevance are widely debated.^15,36–40^ In general, no single model of ischemia appropriately captures all aspects of human ischemic stroke; all have advantages and limitations. Both human and animal studies describe movement deficits contralateral to the stroke damage,^41–43^ loss or disruption of motor and somatosensory representation,^44–45^ loss or disruption of evoked electrophysiological activity,^46–47^ reductions in cerebral blood flow,^48–49^ and cortical infarction.^50–51^ Our particular ischemic method, pMCAO, models a serious (permanent or prolonged) occlusion of the MCA affecting only the cortex. After pMCAO, we describe physical disability, loss of functional representation, reductions in sensory-evoked subthreshold and suprathreshold activity, and severe reduction in MCA blood flow—hallmark attributes of ischemic stroke.^7–8,28^ Despite the advantages of our pMCAO model, which induces ischemia specifically within the cortex, whether the findings presented here may be extrapolated to other ischemic models with different levels or site(s) of injury remains an open question. In the absence of treatment, however, our pMCAO results in an infarct affecting a large cross section of the lateral cortex and in the aforementioned deficits associated with ischemic stroke.

On the basis of recent findings, occlusion of the MCA in a manner very similar to our own also has been adopted successfully in a nonhuman primate study of stroke. Cook, Teves, and Tymianski^52^ demonstrate that surgical clip occlusion of the MCA at the M1 segment can be used to successfully model ischemic stroke in the nonhuman primate and also to evaluate candidate neuroprotective agents (in their case a PSF-95 inhibitor). In agreement with our own and other previous studies conducted in the rodent cortex,^8,53–54^ Cook, Teves, and Tymianski^52^ concluded that collateral blood flow back into the MCA can be a crucial aspect to neuroprotection of the ischemic brain. These groundbreaking findings establish that structural and functional neuroprotection is possible in the higher-order primate brain, and they constitute a major step forward in the process of translating preclinical experimentation into effective clinical treatment.

Although there was a strain difference between our young and aged rat models, on postmortem examination, the different strains sustained similar infarct as a result of pMCAO. Untreated Fischer rats sustained cortical infarct averaging 27.6±2.4 mm^3^, and we previously reported that untreated Sprague Dawley rats sustain very similar cortical infarct volumes of 28.4±2.4 mm^3^.^8^ The infarct data presented here indicate that pMCAO places the Sprague Dawley and Fischer 344 rat cortices under similar amounts of ischemic duress. Although macrophage invasion of the ischemic territory can have the effect of masking the true extent of infarct sustained when tissue is examined as early as 36 hours after pMCAO,^10^ our histological results are in line with previous studies that also have found equivalent infarct volumes between Sprague Dawley and Fischer 344 rats after MCA occlusion.^55^ In the present study, treated aged subjects were protected from the previously described infarct as their young adult counterparts had been in our previous study.

Protection from the described infarct after pMCAO was accompanied by protection from sensorimotor impairment in treated aged subjects (as observed previously in young adults). In addition to causing infarct in untreated animals, pMCAO has been shown previously to impair performance on all 3 behavioral tests used here.^15–16,56^ That treated subjects consistently displayed fully intact performance on all tasks a week after pMCAO demonstrates that stimulation treatment confers lasting protection of sensorimotor capability in an aged animal.

A notable feature of single-whisker-stimulation treatment in aged animals was that it resulted in a recovery rate of cortical function equivalent to that observed in young adults. The similar rate of recovery between young and old rats is clinically important because in both humans and rats, aging results in decreases in basal blood flow, vascular density, collateral vessels, and interarterial connections.^4,57^ We previously demonstrated that the mechanism underlying protection by intermittent whisker stimulation is blood flow reversal in the MCA via collateral vessels and that this collateral blood flow is essential to the neuroprotection described here.^8,58^ The finding that the aged cortex recovers cortical function after an equivalent amount of treatment time suggests that, despite the age-associated degeneration of the vascular system, this protection is not dependent on an abundance of arterial density or collateral connections (most typically the sole province of the young adult cortex).

One difference in ISOI between young and older adults was the finding that the area of the overshoot tended to be smaller in older subjects than in young adults at baseline. Because the overshoot phase of the WFR most typically is associated with evoked increases in blood flow and volume,^59–62^ this observation may reflect the previously mentioned age-related reductions in vascular density and blood flow. Further research is required, however, to definitively confirm that an age-related difference exists in the basal state, given the strain differences (Sprague Dawley versus Fischer 344).

One other difference observed between young Sprague Dawleys and aged Fischer animals was a higher rate of death after pMCAO (≍20%, see Methods for details) among aged Fischer subjects, regardless of group, than that among young adult Sprague Dawleys (<10%). This, however, echoes previous studies in which an increase in death rate in aged compared to young adult experimental stroke groups was observed, and we suspect that age rather than strain accounts for this increased death rate^63^. Despite the ischemic challenge caused by pMCAO, however, the surviving 80% of treated aged subjects did not sustain ischemic infarct.

In conclusion, it is widely recognized that aging negatively affects susceptibility to and recovery from ischemic stroke. In response, numerous studies have emphasized the importance of incorporating aged animals into preclinical stroke research.^4,32–35^ Despite these recommendations, relatively few studies have compared the efficacy of candidate neuroprotective treatments in both young and older adult animal models. As a result, the lack of success of hundreds of potentially promising therapies may have been due, among other things, to an inadequate modeling of the factors that contribute to stroke susceptibility and outcome. Despite the compromised nature of the aged cortex, our findings indicate that sensory stimulation treatment immediately after pMCAO initiates a process of functional recovery equivalent to protected young adult counterparts, resulting in intact sensorimotor capability and a lack of infarct. An observer blind to the ages of the protected animals would not be able to discern which were young adults and which were older adults in terms of the degree of protection. These findings demonstrate that the aged cortex is capable of a surprisingly comprehensive and rapid cortical recovery in response to stimulation treatment, and they encourage further research into similar treatments in human stroke patients.
